# Smallpox and immunisation policies in Argentina from the nineteenth to the twentieth century

**DOI:** 10.1017/mdh.2023.3

**Published:** 2022-10

**Authors:** María Silvia Di Liscia

**Affiliations:** 1 Universidad Nacional de La Pampa, Santa Rosa, Argentina; 2 Universidad Nacional de Educación a Distancia, Madrid, España

**Keywords:** Argentina, Social history, Smallpox, Vaccination, Immunisation

## Abstract

This work examines the history of smallpox, a highly infectious and epidemic disease, in Argentina, throughout different governments and public health policies from the nineteenth to the twentieth century. The study focuses on the smallpox vaccine and the social and collective significance of universal immunization. It also analyses the relationship between governments of different political orientations and the international community regarding the production of vaccines and vaccination campaigns from their implementation to the eradication of the disease.

## Introduction

Smallpox, caused by the *Variola Major* Virus, is the only infectious disease to have ever been eradicated. The eradication of smallpox means a landmark achievement in global public health made possible by the concerted efforts of international organisations [the World Health Organization (WHO) and the Pan American Health Organization (PAHO)] and the participating States. This success has been proudly described by these official bodies and critically examined by a profuse and recent literature.^1^ Argentina is one of the countries of the region supporting this event, as evinced by the country’s mass vaccination campaigns and a subsequent epidemiological surveillance system.

In this article, I explore the long-term practice of smallpox vaccination in Argentina, considering it as a public health policy but also as a technological process that involves, *inter alia*, the medical communities. Hence, I offer an overview on inoculation and variolation, from the first steps of vaccination to the extensive efforts made to expand this practice through mass campaigns at the end of the twentieth century. Such an analysis covers both national and global history, by considering the views of the scientific periphery as well as decisions made beyond national borders that impinged on the country’s public policies.^2^

This historical review enables a close examination of smallpox and its preventive measure, the vaccine, without disregarding its influence on hygiene and health, or the positions of leaders and enunciators, nor the concomitant creation of agencies and their functions. Special attention is paid to the disease-immunity polarity from the end of the eighteenth century to the end of the twentieth century, and how it changes as society evolves. During the course of this period, the territory that later would be called the Argentine Republic (or simply Argentina in this text) underwent significant transformations – from an area dependent on a metropolis to a country with its own forms of government – and the difficulties of a state in economic expansion under an ideologically conservative regime. In the turmoil that was the twentieth century, new actors emerged from mass parties (*radicales* and *justicialistas*), advocating for an industrialist and interventional socio-economic system. During this period, popular decisions were restricted and censored by several *coups d’état*, under the authoritarian military and corporate administration. Furthermore, important demographic transformations took place: life expectancy at birth increased from 33 to 75 years between 1895 and 1990 and the death rate decreased.^3^ The illiteracy rate also declined, in line with the compulsory primary education established in 1884: in 1869, it was 71.4%, and, in 2001, it was 2.6%.^4^

The Argentine case, therefore, provides important insight into how vaccination was established to control a serious disease and the ways in which these efforts coincided with the interests of the medical elites close to the State and influenced the decisions of international organisations. The study explores the dissenting views concerning inoculation and vaccination practices, based on potential accidents and interference with the human body, that is, the individual interests in conflict with the advantages of an immunised community.^5^ Attention is also paid to the disfiguring bumps smallpox caused on the body of an infected person: a few days after the initial infection, the bumps develop into fluid-filled lesions and then into painful, pus-filled ones. This sparked fear around the world, and doctors spoke of smallpox as one of the ‘ugliest and most horrific’ ailments.^6^

Vaccination, the main focus of this work, is linked to State research, production and dissemination strategies for both the product and the health messaging of immunity addressed to the target population. Different studies in other Latin American nations have also focused on these themes, especially on mass vaccination campaigns.^7^ Lastly, this paper considers the sanitary-political aspects involved in eradication, from the WHO–PAHO resolutions of 1958 onwards. An examination of international campaigns reveals the leading role of other actors and the intent of several nations to negotiate on equal terms.^8^ In the particular case of Argentina, these aspects shed light on worldwide discussions on the spread of epidemics, knowledge, medical and scientific communities and sanitary control measures.

The sources (published and unpublished texts) drawn upon to write this paper include medical theses, manuals and publications of the Departamento Nacional de Higiene (DNH) and other official journals, such as those of the Consejo Nacional de Educación. The digital repository of the PAHO and the WHO between 1920 and 1988 and the press in general were also consulted.

## Inoculation and variolation: successes and failures

Smallpox pandemics came to America with the arrival of Europeans, and although it is difficult to accurately determine their demographic impact, these events undoubtedly affected Western control over the original ethnic groups, as they implied a strange and highly contagious disease related to the groups in power.^9^ No palliative was known, and, in addition to fatalities, the epidemics left permanent marks on the skin of the victims and sometimes even blindness. At the end of the eighteenth century, today’s Argentine territory was under colonial control, and inoculation practices were already common among the ‘white’ population, that is, descendants of Europeans. Inoculation was an ancient practice used to immunise individuals with material taken from a recently infected person, in the hope that a mild, but protective, infection would result.^10^

The experiments carried out by Edward Jenner on the cowpox virus – similar to smallpox – are well known, as well as the Balmis Expedition, aimed at vaccinating millions (including the rest of Europe and America) against smallpox between 1803 and 1804. These initiatives resulted in practices which, though related, were not exactly vaccination. Some people (not always doctors) used different methods to spread the ‘implanted’ virus: a Portuguese slaver, for example, in 1805, brought the vaccine to the Rio de la Plata on the arms of enslaved people who had been already vaccinated.^11^ In 1810, the colonies became independent from Spain. One of the few health measures in those turbulent times was the so-called *beneficio vaccínico* (‘vaccine inoculation’). It was applied on the arm, although women were recommended to get it on the thighs to avoid the horrible disfiguring rash, which could dissuade them from receiving the vaccine altogether.^12^ Another opposing argument (though more reasonable) was the possibility of contracting serious diseases (other than smallpox) from those who had been infected with the virus. For decades, this preventive measure was resisted by ‘the common herd’, as doctors would say, and called for substantial public efforts.

In the second half of the nineteenth century, a group of medical professionals interested in applying microbiological theories to health practices made their way into the public system, fostering an institutional reorganisation. Despite the persistence of this medical community, their proposals for mandatory vaccination, submitted from 1871 to 1886 in the Province of Buenos Aires and later in the Federal Capital and National Territories, were not approved.^13^ The rejection can be attributed to the power doctors exercised in certain jurisdictions, especially Emilio Conti and José Penna, and their political influence on the legislative bodies, rather than to specific technical reasons. In the end, a concern for contagious diseases and, more specifically, the epidemics among indigenous people – who were brought to Buenos Aires after the extermination campaigns – provided further justification for the vaccination proposal, and the measure was finally accepted.^14^

In 1881, Louis Pasteur coined the term ‘vaccine’ (in honour of Jenner) to refer to the artificial substance used to prevent rabies. Those studies quickly became known at the local level, giving vaccination a greater scientific weight. Almost at the same time, however, the International Anti-Vaccine League emerged in France, holding congresses in different European cities.^15^ In 1853, a similar organisation was established in Great Britain, which fought to limit the vaccine in defence of liberal values, and denounced it as an authoritarian intervention on the popular sectors; in 1907, the organisation obtained victory through ‘conscientious objection’.^16^

In 1876, vaccination became mandatory for immigrants by virtue of National Law No. 817, and, in 1880, it was also made mandatory for schoolchildren pursuant to National Law No. 1420. But it was only in 1886 that smallpox vaccination was made compulsory for the entire population of Buenos Aires, and in 1903–4 for the Federal Capital and National Territories, as we will see later on.

Towards the end of the nineteenth century, Argentinean individuals and families expressed their discontent with and reluctance to vaccination, although it was not an organised resistance, likely because a large group of intellectuals and other professionals linked to the State supported doctors’ ideal of a secular state. The opposition can be divided into two large groups: on the one hand, the popular sectors, who held a fatalistic world-view and were fearful of accepting a remedy that produced the disease itself (albeit attenuated), and on the other hand, professionals and civil servants, who were reluctant to implement a presumably ineffective, inconvenient and costly measure. It is important to mention that, at this time, the debate did not go beyond the limits of the Province of Buenos Aires, although it was reasonable to propose similar measures for the rest of the provinces, to formulate a nationwide programme was unthinkable due to political reasons. The population’s refusal to freely accept the measure added to the difficulty of isolating infected patients and preventing contagion, an effort which relied heavily on the beliefs and customs of the popular sectors and the Church.

Doctor Lucio Meléndez’s account of the first major epidemic, which occurred in 1871, revealed these and other difficulties; the doctor claimed that even though the epidemic was a fatal event, ignorance and apathy had fostered its spread. The ‘deadly disease’ circulated from the northern towns of the Buenos Aires province (San Pedro, Junín, Baradero and Zárate), along the coast of the Paraná River, into the province of Santa Fe and the port of Rosario. Filled with indignation, Melendez wrote to his colleagues that he had never found greater resistance on the part of adults and parents to receive the benefits of the authentic and incontrovertible finding made by Jenner. Some refused to get vaccinated arguing that they were not certain of the quality of the smallpox they would be receiving; others claimed that the prophylactic vaccine was useless: if it was decided by God that their children should get infected with the scourge and eventually die, everything was pointless. Having authority to speak in the name of science and based on centuries-long experience, the doctor was sadly disappointed: he was getting a ‘no’ grounded on illogical reasoning, on primitive and unsubstantiated ideas [sic]!^17^

However, vaccination was not a once-and-done event; it involved several attempts, and most often the vaccinated individuals would not return for the doctors to check for the pustule indicating a positive immune reaction. Therefore, in the partial statistics on vaccinated people in Buenos Aires between 1878 and 1886, positives ranged from 37% to 59%, and negatives from 17.4% to 8.1%. The unknown cases ranged from 45% to 33.4%. Once the vaccine was made obligatory, the first move was home vaccination across Buenos Aires neighbourhoods. Medical doctors argued that Creoles and immigrants, recently settled in very poor tenements and not having enough knowledge, did not comply with revaccination or refused the benefit altogether.^18^

As we shall see in the next section, public institutions made it possible to extend vaccination across a large part of the national territory.

## An immunised national community

In 1880, the National Congress approved by law the organisation of the National Department of Hygiene (DNH), with jurisdiction over the Federal Capital and the National Territories; one of its aims was to fight epidemics. In the ‘historical’ provinces, there were Hygiene Councils (Consejos de Higiene), and in the Capital City (Buenos Aires), the municipal government organised the Public Assistance (Asistencia Pública). Smallpox vaccination was one of its main objectives.^19^ The DNH was presided by distinguished physicians with a solid political profile: José María Ramos Mejía, Eduardo Wilde, Carlos Malbrán, José Penna, Gregorio Araoz Alfaro and Miguel Sussini.^20^ In 1913, the central government through the DNH organised the Public Assistance in the National Territories with similar hygienic purposes.

Vaccination opened new horizons. Doctors and other specialists already knew techniques to obtain the product from animals with similar viruses, as well as methods for preserving the lymph, and a virus attenuation system to reduce risks. Before the creation of the Official Preservation Commission (Conservatorio oficial), the vaccination context was quite bizarre, with the animals accompanying the vaccinator in their task across the different homes. Vaccine manuals and theses thoroughly indicated the positions of the doctor and the patients and how to take the lymph from the bovine pustule and introduce the virus into the human organism.^21^ The indications described an artisanal practice, quite similar to inoculation.

By generalising vaccination and extending the measure with mandatory coverage, the scale of development also changed and the public health system organised ad hoc institutions. In the case of Argentina, since 1890, the entire production had virtually concentrated in the Conservatorio Nacional de Vacuna and the Instituto Veterinario de Santa Catalina in the Province of Buenos Aires. By 1901, vaccination was carried out at the Instituto de Bacteriología in the Capital City of Buenos Aires, renamed Instituto de Bacteriología, Química y Conservatorio de Vacuna Antivariólica (Institute of Bacteriology, Chemistry and Preservation of Smallpox Vaccine) in 1916.^22^ The Public Assistance, as well as other hospitals and health centres, had a Bacteriological Laboratory for routine examinations that were used to verify the product.

Alfredo Larguía detailed the procedure to produce vaccines with the Cow Pox method, from how to choose the ‘vaccinogen’ (Hereford or Durband calves), to how to wash and inoculate the virus in the abdominal region, and how to collect the pulp to ensure an antiseptic preparation, as vaccination could produce new pathologies.^23^ One of the main reasons for popular reluctance was the well-known transmission of serious diseases such as syphilis and tuberculosis or gangrenous infections through contaminated vaccines.

Vaccine lymph was mixed with glycerine to prevent decomposition and placed on ten glass plates. The animal was destined for necropsy to certify its health and the existence of the Cow Pox. Two of the plates in this series went to the Bacteriological Laboratory to certify the absence of other microorganisms; two other plates were used for vaccinating a number of people at the Institute. The vaccines were ‘pure’ (without bacteria), but could be inert, and consequently, the strain of that series was to be discarded as it had no immunising effect. If they tested positive, plates were prepared for nationwide distribution. The next step was preservation: a delicate task that required extreme care, since the glycerinated and dried lymph was placed in plates at 120ºC for half an hour and then covered with paraffin to keep it preserved for 3–4 months in a cool place. Each plate was tagged with the name ‘vaccinogen’ and the date.^24^

After production came distribution, and to the regret of those in charge, there was too much waste. The provincial Hygiene Councils received packages of up to fifty plates by mail. Between 1875 and 1901, 2 106 070 plates were distributed to vaccinate more than 4.5 million people, almost the country’s total population. But the public institutions only registered 323 000 people vaccinated, and of them, 231 947 were reliably immunised, 12 615 showed negative results and 24 758 were unknown. The Asistencia Pública of Buenos Aires could have vaccinated twice as many people, and the same can be said of the provinces of Santa Fe, Córdoba, Tucumán and Mendoza. Plates produced with extreme care were going to waste, and private institutions were taking advantage of what ‘the State provided for free.’^25^ The main problem seemed to be distribution: less than 10% of the shipment was used, and inappropriately. The whole batch would have been enough to achieve universal immunisation at an early stage.

The epidemics did not cease, and the State was not entitled to prohibit the entry of unvaccinated immigrants or to vaccinate children in their homes without parental permission. The decision to visit the official centre and get the vaccine fell on the population itself. There was partial commitment on the part of provincial authorities and even the City of Buenos Aires, where the Public Assistance was supposed to set the standard for medical care at the time. But the scene changed. Larguía’s director was José Penna, who had also dealt with smallpox in his doctoral thesis. Penna’s dazzling career includes the leadership of the Public Assistance in 1906 and that of National Deputy between 1910 and 1914, in addition to being president of the DNH until 1916.

The work of both Carlos Malbrán (also a national Senator) and Penna as heads of these frontline institutions reinvigorated the health system as a whole. Vast resources were obtained from Congress. Thanks to these sums, systematic vaccination campaigns were organised, first in the Buenos Aires neighbourhoods and then in the National Territories, from the beginning of the twentieth century. The DNH expanded vaccination to foreign immigrants in the capital and to residents of other provinces, especially in schools as the practice was mandatory for admission to educational institutions:

Vaccination is unquestionably the most accurate, most perfect and safest prophylactic form and at the same time the easiest to implement in order to provide immunity (…) Based on the results obtained for smallpox, artificial immunization through vaccination cannot be surpassed by any other preventive means; the effectiveness of vaccination is certain and positive, and this method may achieve the extinction of epidemics. Isolation, the sanitation of infected sites, and disinfection are inferior practices.^26^

In 1912, Penna put forward a Bill to make the vaccine obligatory in the entire national territory, but it had to be approved by the executive branch in concert with the provincial governments.^27^ The law was not passed, and the law 4202 of 1903–4 remained in effect in all the national territory. The historical provinces did not pass any other laws to replace the law in force in the National Capital and National Territories, and throughout the twentieth century, they followed that national regulation.

The parallel expansion of public education aided the process: this pedagogical and medical interplay helped to consolidate the ideal of a modern nation. The Cuerpo Médico Escolar (School Medical Corps), belonging to the National Council of Education and formed in 1884 by the medical elite, was in charge of certifying vaccination to enter and remain in the public health system. Publications promoted the advantages of universal immunisation as well as the different campaigns and methods of vaccine production.^28^

The organisation of campaigns – including itineraries, vaccinating agents and coordination with railways and vaccination venues – probably represented most of the work carried out by the DNH Vaccine Office, presided by another prominent hygienist, Nicolás Lozano. The health policies of the time were encapsulated in these ‘portable institutions’ seeking to reach all corners of the Republic. Health agents, generally trainees or auxiliaries, would travel through deserts and crossed mountains and rivers to get to unknown towns and complete vaccination of whole families, with the assistance of local policemen and teachers.

Smallpox mortality decreased considerably; the few works available state that smallpox had disappeared in a short period: between 1911 and 1914, there were 4 420 deaths. In 1911, 4 024 deaths were registered, and only 17 in 1914. At that time, mortality due to other diseases, such as bronchopneumonia, was much higher (24 725 deaths).^29^ Yet, it is important to note that for the latter disease, there were no palliatives (antibiotic therapy would appear later on), whereas for smallpox, the vaccine was indicated as the only possible solution.

In 1921, Argentina produced 1 967 770 doses, for a country with more than 8 million inhabitants. Almost one hundred people worked in the production process at the aforementioned Institute, which served as a distribution centre for the whole country. That year, the Bacteriological Institute used 627 calves and obtained 25 361g of lymph for the almost two million plaques.^30^ This was one of the few successful national policies that the country could be proud of as it involved a methodical coordination from vaccine production to distribution.

Like other countries, Argentina joined the efforts of international organisations concerning epidemic control. Smallpox was one of the diseases registered thanks to the efforts made by the then Pan American Office (later PAHO). In the 1920s, this agency issued monthly publications with statistics on smallpox cases, evincing a steady progress towards the elimination of this disease.^31^ In certain Argentine provinces, the press closely followed these outbreaks and emphasised the need for vaccination.^32^ Only one case was reported in Argentina in contrast with 223 cases – and 131 deaths – documented in Brazil.^33^ In 1928, an exhaustive review by delegates Laurentino Olascoaga, Nicolás Lozano and Alfredo Sordelli before the 8th Pan American Conference revealed that the disease was completely eradicated. Other promising indicators, such as the decline in infant and general mortality (16/1 000 and 116/1 000, respectively), made Argentina the Latin American country with the best demographic prospects.^34^

According to the available data, in the 1930s, campaigns were developed in the southern territories during the summer, reaching inhospitable areas in winter. The Head of the Vaccine Section indicated that 670 245 plates were distributed, covering 127 790 people throughout the country, and that 319 072 people were revaccinated. Immunisation was not yet guaranteed since the population had to be re-vaccinated periodically.^35^ In addition to the vaccine, DNH at certain times imposed restrictions on the movement and isolation of those infected, measures difficult to maintain for a long period. However, when a smallpox epidemic was declared in Entre Ríos, fearing that it would expand to Santa Fe, the authorities activated a vaccination and revaccination operation along the coast and prohibited access from neighbouring provinces.^36^

Vaccination became a routine practice but was relegated when other emergencies came up, such as campaigns and projects to control malaria, plagues, typhus and hookworm.^37^ In the thirties, both the middle and popular sectors pressed with numerous demands and demands in the face of unmet social and health problems, in pursuit of a mother–child agenda and the general perception of a health crisis. People from different political and professional sectors (including Medicine and Social Sciences), who belonged in the progressive and conservative spectrum, registered an increase in cases of social diseases (tuberculosis, syphilis and leprosy, among others), as well as a decrease in birth and fertility.^38^ Smallpox became a controlled but not completely eliminated disease (an increase in cases was reported in the mid-1930s). In 1936, there were eighteen cases in the *yerba mate* plantations (*yerbatales)* in the Province of Misiones, bordering Brazil; the infected workers were isolated, and 250 000 people had to be vaccinated (or revaccinated).^39^ A year later, infections were also reported in Corrientes, Misiones and Entre Ríos, as well as 349 cases in Jujuy (Argentine–Bolivian frontier), attributed to the arrival of sugar harvest workers, who moved from one country to another.^40^ In the case of the northern jurisdictions, DNH mentioned that the contagion was due to the migration of labour from the neighbouring country, and to ‘alleged failures’ in vaccination, since the plates could not be kept in optimal conditions.^41^

Another outbreak in Punta Alta, a city of the Province of Buenos Aires, led to a forceful measure: the then de facto President issued a Decree to establish mandatory vaccination for all national and municipal public employees. In addition, any person applying for bureaucratic procedures would be required to show the Certificate proving that they were immunised.^42^ Previously, only immigrants and schoolchildren were asked for this certificate; the measure, then, had been broadened to cover a larger group of adults, who were presumably already vaccinated.

The Institute of Bacteriology referred to above produced new vaccines, such as anti-rabies; serums, such as anti-tetanus and anti-venom and other biological products, such as insulin. However, these products were not as massive and widely distributed as the antivariolic vaccine, of which the Institute manufactured hundreds of thousands of doses.^43^ Vaccination policies were conceived from a state perspective, with vaccines being produced in series by a public institution following methods that were kept almost intact until the 1970s.

In 50 years, Argentinian vaccination techniques had changed very little and the production process had not seen any significant changes either. Since 1885, the first generation of scientists receiving the legacy of Pasteur had explored different ways of attenuating viruses through high temperature, chemical agents and the use of certain strains or cell culture, which enabled the manufacture of viral vaccines of considerable technical and scientific relevance starting in 1949.^44^ In the mid-1930s, the production of vaccines was (according to the agency in charge) more efficient as fewer people produced more doses. Alvarado, a recognised public health official in charge of this section, indicated that, in 1936, thirty employees of the Institute had managed to obtain 55 319g of lymph and produce 2 528 530 doses out of 222 calves.^45^ Lymph containing the cowpox virus was mixed with glycerine as in the past, but other features had been improved, such as the preservation and duration of immunity. These were extremely important for determining new strategies: lower volumes were needed, and a different transport system was required. For example, the ‘dry vaccine’ tested at the Institut Pasteur in Paris and another city performed better than the wet ones because it was easier to distribute, and studies conducted in Germany and the United States on the immunisation unit obtained from the bovine lymph (used in Argentina) showed that it would lose its effectiveness within 2 years, which made permanent revaccination an obligatory requisite.^46^

The national objective was to consolidate centralised public policies and guarantee production, preservation and official distribution with universal coverage, at the cost of creating and maintaining a system with a low degree of innovation. One of the few improvements with regard to lymph preservation was the replacement of vaccine plates with capillary tubes and collapsible tubes containing from twenty-five to fifty doses, when the Vaccine Section became fully integrated within the Institute of Bacteriology.^47^ This agency had been the spearhead of biological and physiological research (first led by Rudolf Krauss and then by Alfredo Sordelli) later paused by the steady production of serums and vaccines.^48^

Between 1945 and 1955, a sequence of important changes took place in the national health scenario, the antecedents of which may be found in state intervention and public management policies since the 1920s, introducing concepts such as the ‘right to health’. Among the campaigns developed, smallpox did not have the priority of old times, which is why outbreaks reappeared around 1949.^49^ There were debates as to whether the virus was that of smallpox or alastrim, a disease with less serious effects on the human organism.^50^ The 365 cases had a shocking impact on the vaccination system: the word was spread that immunisation was unsafe as the material was being refrigerated for over 24 hours; this uncertainty invalidated the process as a whole and raised again the dilemma of which product to use and how to preserve it properly for distribution.

In 1950, it was said that the ‘showcase vaccine’ was to be blamed for the return of the disease; this vaccine was kept without refrigeration, and did not seem to protect against infections. Furthermore, vaccination campaigns had declined.^51^ The National Council of Education launched the school health system to promote (again!) the advantages of vaccination, and the person in charge of vaccinating was supposed to explain in detail the manufacture of the vaccine and to assuage the population’s fears on hygienic conditions and preservation measures, emphasising the use of storage chambers between 4°C and 8°C for a 3-month period, and dried vaccines only under special conditions. A century later, the advice that ‘in the female sex the application on the thigh is preferred, to avoid the appearance of ugly scars’ was still commonplace.^52^

In 1957, only 40% of the population was reported as immune, according to national authorities.^53^ This concern prompted mass vaccination campaigns, and a new vaccine was launched in the north of the country, produced by the Instituto Nacional Carlos Malbrán and the PAHO.^54^ The campaigns were an initiative of international organisations, which local health administrators were able to enforce. The way was being paved for the eradication of the disease (examined in the next section.)

## The eradication of a disease

More than 30 years elapsed from the first deliberations of the WHO on the problem of smallpox (1948) to the effective eradication of the disease in 1980. Backed by the PAHO, the United States launched specific programmes in America starting in 1949, but in the mid-sixties, after realisation that little progress had been made, efforts were intensified.^55^

The relationship between eradication campaigns and the Cold War is interesting since decisions were made based on the organisational and technical support of both powers – the United States and the USSR. Latin America, however, was one of the protagonists in the *Encuentro de Punta del Este* in 1961 and would then provide new insight into the actors and themes of inter-American relations.^56^

Two years after this summit, the Health Ministers of the Río de La Plata countries, under the auspice of the Inter-American Development Bank, instituted declarations on the control of communicable diseases. Smallpox had a special place in these 1963 conversations between high-rank officials from Argentina, Brazil, Paraguay and Uruguay, aimed at coordinating support campaigns across borders. Specific measures included the promotion of diagnostic services, meetings between campaign managers and increased production of good quality ‘freeze-dried vaccine’ to replace the vaccine prepared with glycerine.^57^

In Argentina, law No. 15465 of 1960 established the obligatory reporting of ‘Group A’ infectious diseases such as smallpox and alastrim (considered as a different virus). Health professionals – including physicians, veterinarians, pathologists, obstetricians, dentists and kinesiologists – were all under obligation to immediately notify the nearest health authorities of any cases (or suspected cases), even if they were made aware of these by third parties. This measure brought smallpox back to the fore. Previously a neglected disease, with outbreaks that called for urgent (often improvised) measures which immediately fell into oblivion, it was now considered a dreadful scourge. Thus, the requirement to report data on possible infections from anywhere in the country and even before laboratory tests were carried out (required for reporting diseases of Groups B and C) meant that the government had scaled up actions to deal with this disease, classified as Group A.^58^

In 1958, when the plan for the eradication of smallpox was relaunched by international organisations, the country had already expanded scientific research and was planning to establish institutes of sciences (later cut short by the military coup of 1962.)^59^ Building on this momentum of scientific development, the Instituto Malbrán was refounded with many biomedical projects oriented to genetic research. The institute appointed renowned scientific personalities such as Ignacio Pirosky, purchased state-of-the-art equipment and reinforced training through scholarships for studying in laboratories in Great Britain and France, two countries excelling in this new field, biomedical and genetic research. The democratic breakdown of 1962 stifled this growth that might have driven Argentina through important research roads; a clear example of this is the exile of César Milstein, who was impeded from continuing his work in Malbrán.^60^ From then on, the Institute readopted its previous ‘sanitarist’ approach, focused on epidemiological control and traditional guidelines.^61^

The obligatory reporting of 1960 remained in force and is still applicable today for COVID-19. In Santa Fe, littoral province of Argentina, for example, campaigns promoted vaccines applications with the ‘very pure serum provided by the Institute of Microbiology’ (Malbrán), which were refrigerated to prevent them from spoiling.^62^ Furthermore, in this interval of democratic government, the campaigns implied home vaccination, a community intervention approach and a provincial scope. In the capital city, with 208 900 inhabitants, fifty-two brigades were formed, aided by unions, corporations and student councils, and refrigerated chambers borrowed from stores, schools and individuals were employed. The whole population of the city was vaccinated, and monitored in case revaccination was needed; a certificate was issued accordingly. This campaign, profusely covered by the press, was then carried out at the provincial level, with supplies provided by the nation pursuant to WHO regulations.^63^ The province was one of the three most populated provinces in the country, with a total of 1 884 918 inhabitants.^64^

Following an expanded production of the smallpox vaccine (mainly after 1967), Argentina’s efforts for the smallpox eradication largely involved mass vaccination campaigns. The government, however, did not employ any of the strains recommended by the PAHO and the WHO. And until 1967, despite international recommendations to use lyophilisation, Argentina continued employing the glycerinated vaccine. The reports of the different nations participating in the eradication programme subtly reveal that despite the WHO and the PAHO initially requested that only freeze-dried vaccine be used, the Argentinian government negotiated the massive use of the glycerinated version, which was more economical, at least from the first stage until the intensified worldwide programme of smallpox eradication.^65^

The 1958 decisions on the eradication of the disease did not translate into concrete actions until 1967, when the WHO required member countries to create a Fund to speed up the process. With the ridiculous amounts contributed by Argentina, no significant changes could be made: while the United States contributed US$ 26 241 403, Argentina invested a modest sum of US$ 13 275. This difference may be due to the fact that the world power was also one of the members of the Security Council and a major representative of its policy; compared with other Latin American nations, Argentina’s amount was still very small as Brazil had contributed US$ 128 925 in the same period.^66^

Between 1967 and 1980, Argentina implemented the intensified eradication programme, which also included innovative genetic studies on Orthopoxviruses to look at possible animal reservoirs and anticipate future infections.^67^ Additionally, a survey covering seventy-seven laboratories in fifty-two countries was conducted to assess the quality of the vaccine; as the answers were grouped by continents and not by nations, there are no separate data available for Argentina. What is known for certain is that the Argentinian laboratory actually responded the request for comments on the production, conservation, distribution and other technical aspects of the vaccines, unlike other Latin-American countries (such as Bolivia, Ecuador and Peru).^68^

The PAHO and the WHO had to certify the quality of vaccine batches, as vaccine production was far from flawless, especially in South America, Africa and large parts of Asia. In Argentina, as mentioned before, a single laboratory produced all the vaccines, and then the Connaugth Laboratories in Canada conducted tests for viral potential, stability and degree of bacterial contamination. Members of the PAHO and the WHO (Canada, the United States, Great Britain and the USSR) would participate in seminars and meetings held by experts (eg. USA – 1968); however, no country from Africa, or other Asian or SouthAmerican countries, became involved. Specific indications were given on how to keep vaccines in optimal conditions with refrigerated equipment and how to effectively use them at the moment of vaccination.^69^ Although Argentine professionals, like others from all over America, received training from experts and became familiar with the most innovative procedures, they did not become leaders in any of the production phases.

Argentinian batches were evaluated as ‘satisfactory’, and 810 000 doses were earmarked for the intensified campaign; a comparable number was obtained from other South American countries such as Peru and Brazil.^70^ Until 1967, lancets, the vaccinostyle or simply a needle was used; in 1969, the bifurcated needle was introduced, recommended by the UN for a more effective application and to save doses. Difference strains were used for lyophilised production: in Argentina, the Massachusetts 999 strain was used in 1968, whereas in 1971 and most probably due to international recommendations, the Lister strain was employed.^71^

At that time, the policies to eradicate smallpox were not a priority for the Argentine nation, given the number of documented cases. Between 1959 and 1971, there were a total of 211 cases, but during whole years, no infections were detected (1963, 1968 and 1971), and other years (1967), only patients who came from nearby regions were treated. The permeable border with neigh boring countries explicates the persistence of cases on both sides; for example, in 1970 in Rio Grande Do Sul, a young woman who came from Colonia Alicia (Misiones Province, Argentina) got infected and triggered vaccination or re-vaccination of 84% of the provincial population.^72^

Compared with countries with regions where the disease was endemic, such as Brazil, Colombia and Ecuador, Argentina’s epidemiological situation was significantly better, as smallpox was no longer an uncontrolled disease. Nonetheless, in order to comply with the international obligations, measures had to be implemented with a national budget (Argentina received amounts for the programme from both the WHO and the PAHO); between 1953 and 1971, the country received US$ 236 000 from a total of US$ 1 124 000, of which, Brazil was the principal beneficiary.^73^

Yet, although very few cases were documented, the vaccination actions were undoubtedly remarkable, having almost a universal scope. The campaigns carried out in the intensified phase, between 1967 and 1972, virtually encompassed the entire South American territory. In the Argentine case, only the Provinces of Buenos Aires, and part of Neuquén and Entre Ríos, were excluded from the mass vaccination programmes.

Rodrigues, head of WHO South America and Adviser on Smallpox Eradication, indicates that hundreds of thousands of people were vaccinated annually in that period, when Argentina had more than 23 million inhabitants. Thus, in 1967, 1 808 000 people were vaccinated; a year later, the number was 324 000; in 1969, 2 141 000 and in 1970, the figure rose to almost half the population: 11 009 000 people.^74^ Following the century-old tradition, the vaccines were produced in the country, with the new technology suggested by international organisations. The Argentine effort was remarkable; 560 000 were manufactured in 1967; in 1968, 14 944 800; in 1969, 21 427 850; in 1970, the figure doubled to 44 350 325 doses; in 1971, 12 218 600 were produced and a year later, in 1972, the number was 17 456 000.^75^

The intensified smallpox eradication programme was followed by the epidemiological surveillance programme; in Argentina, this programme relied on the data obtained by virtue of the aforementioned law requiring that suspicious cases were immediately notified via telephone or radio communication. In 1971, consultants hired by the PAHO made visits to fourteen locations in the provinces of Buenos Aires, Corrientes, Misiones and Santa Fe and conducted eighty-six surveys; no smallpox infections were detected.^76^ In 1973, new massive campaigns were carried out, urging the population to exercise their right, and stressing at the same time that it was a community duty. The vaccines were now freeze-dried and more effective, preserving live viruses without refrigeration; thus, the ‘flaws’ of glycerinated vaccines were no longer an issue.^77^

Based on the above, and the disappearance of cases in Argentina since 1971, the WHO declared the country free of smallpox, and under the recommendation of the Sociedad Argentina de Pediatría (Argentine Society of Pediatrics), smallpox vaccination was ceased in 1979, in the midst of a military dictatorship, due to the risks it posed to children who had only a remote chance of getting infected.^78^

## Conclusions

In 1980, while announcing Smallpox eradication, the WHO stated that no similar policies would be implemented for other diseases. The question emerges as to whether this initiative was indeed a most urgent priority on the agenda of many underdeveloped or developing countries, which relegated other investment in human resources and health infrastructure, perhaps less urgent, but more necessary. In the particular case of Argentina, it is worthwhile recalling that containment had been achieved much earlier, with a mass vaccination policy aimed at an extensive social group.^79^ The outbreaks that followed were limited to a controlled endemic area. Furthermore, although the basic vaccination technology was derived from experiences outside the country, Argentina effectively applied its own production system at an early stage without requiring support from private laboratories or any other outside help. Such was the scenario at the beginning of the twentieth century, shaped by medical professionals working closely with the State. Some fluctuations though no steps back occurred until the ratifying of PAHO–WHO international agreements for the eradication of smallpox.

One of the issues inviting some reflection is the population’s objection to variola inoculation from the beginning to almost the end of the nineteenth century. Doctors at that time did not have power enough to enforce the measure, which consisted in the artificial transfer of a virus from person to person and implied high risk of smallpox infection and contagion of other diseases. Unprotected people – minors, captive ethnic groups – were used both as a reservoir to vaccinate ‘arm to arm’. Vaccines implied an artisan practice, with little public control and therefore susceptible to deviations from the established scientific canon. After recurrent epidemics, however, a solid medical community linked to the State would assert that the persistence of the disease was a reflection of a weak country with an ignorant population. Smallpox vaccination was then made obligatory.

Around 1904, another period started. Vaccination was enforced by law in some parts of the country and later extended to all provincial jurisdictions. Vaccination with Cow Pox was rebranded as antiviral vaccination, and the DNH centralised production together with the Instituto Bacteriológico. Although sanitary agencies were altered (in terms of composition and scope) along the process, vaccination was one of the few sustained sanitary practices until the elimination of the disease in 1979. The system involved a wide production circuit and included a veterinary sector (of several hundred calves a year), technicians to extract and manipulate the lymph with glycerine in glass plates, and a distribution circuit. Distribution was first by mail, and then, to improve efficiency and enable effective application, vaccination agents were appointed (across the National Territories and the Federal Capital). The initial campaigns were held in the first decade of the twentieth century, and the ‘itinerant practices’, provided the State with some advantages beyond medicalisation such as the acquisition of territories and the confidence of their population, most of which were inhabitants who had just arrived from other countries and regions. More resources became available owing not only to an expanding economy but to certain moves of the political sectors. It can be said that vaccination served as the civilising and sanitary *arm* of the State in recently conquered spaces taken from native ethnic groups, where overseas population were landing.

National educational agencies helped to demystify the popular stigmas surrounding vaccination. The increase in literacy first expanded vaccination against smallpox and later against other diseases, such as diphtheria and polio. In charge of bureaucratic certification, educational agencies would complete the control cycle. However, the hygiene–education pair failed to remove the aesthetic stigmas of the disease, a supposedly greater concern for women (‘weaker sex’). According to gender canons, women should take care of their appearance if they wanted to get married. This is an interest subject for further research, especially in relation to other pathologies which were much more serious but did not cause oozing pustules or leave marks on the skin.

In the twenties and the thirties, the manufacture of the smallpox vaccine became the exclusive responsibility of the public system. The pharmaceutical industry expanded thanks both to the Argentinian market growth (showing an increase in medicalisation within the middle classes) and the political scenario after the bans on imports. However, it did not manufacture the vaccines. This was a task of the State, which produced hundreds of thousands of doses per year and provided them free of charge. The standardisation of the technical process yielded more doses, and plates were replaced by tubes; however, this was not enough to eliminate the disease, as permanent re-updating was required. The system started failing because innovation had not been a priority.

Containment but not elimination of smallpox was achieved; it became an endemic disease with outbreaks every 8, 10 or 12 years; there were cases in 1923, 1935–7 and 1949–50 in different provinces, where vaccination was required once again. At those times, shortage of product was reported in some areas and deficient quality in others, which encouraged technical improvements. No organised resistance was found from any sector or movements against this measure, which – as stated in health announcements – had been made compulsory and universal without distinctions of any kind.

That is why the eradication programme was accepted; Argentina implemented it with a new law (1960) which established the reporting of diseases and implied serious sanctions for health professionals if suspicious cases were not reported in a timely manner. Smallpox acquired a new dimension: first an almost disregarded disease which appeared periodically to remind public policy makers of its existence, it became a possibility to give back the Nation a place in the international sphere. In relation to debates on eradication, however, professionals from Argentina, unlike Brazil, did not have direct participation in the decisions of the WHO nor in the PAHO except as participants in training seminars.

Different sectors embraced the ‘cause’ of eradication, and no tensions were identified despite the fact that the process occurred in the midst of abrupt political changes, under democratic as well as dictatorial regimes. This consensus in pursuit of the smallpox eradication is particularly striking, since agreement was lacking in many other respects. For example, in relation to medical care and medicine supplies, which involved unions and powerful medical–pharmaceutical corporations and the State, it is known that pharmaceutical companies supported sectors opposing the governments in power.^80^ Based on these conflicts of interest among private sectors, unions and professional corporations, a unified health system was unthinkable.

In this context, Argentina managed to set foot in the international scene with a humanitarian and highly promoted measure, which ended up ‘exterminating’ the disease.^81^ During the turmoil of the Cold War and then, with the increasing demands of the Third World, the eradication of a disease meant an incentive to reduce poverty or inequality, a goal as idealistic at that moment as it is now. Along this journey based on the agreement of very diverse nations, the PAHO led the campaign in South America. Argentina showed some reluctance at least in relation to technical matters.

The country received few resources for the programme; it was not an enthusiastic financier either, since contributions were particularly meagre. One of the main campaigns carried out in 1960 at the provincial level mentions ‘national’ aspects and the role of the WHO only to indicate evaluations regarding the type of immunisation practice. Although not recommended, glycerinated vaccines were used. And the viral strains were not the ones used in other regions…. Was it due to a special development in the country or a kind of laboratory atrophy? The Institute in charge of this, which had been producing vaccines for several decades, was modernised and introduced new aspects of biotechnological research in the late 1950s; Later on, however, the previous sanitarist approach was re-adopted during both authoritarian and democratic administrations. This context may explain the fact that the vaccine process produced millions of doses (with annual increase) employing obsolete systems. The material could cover 20 million Argentines, the total population at that time, but quality was not good enough since periodic revaccination was required as in the past. It was not until the 1970s that the more stable lyophilised vaccines were used, most probably thanks to the substantial support given by the WHO.

When was smallpox under control? According to demographic information, this infectious disease ceased to be a health concern around 1920; subsequent outbreaks were related to certain neighbouring or port towns and migrant workers. In the sixties and the seventies, eradication efforts with mass vaccination or revaccination depended on the degree of media impact. Even in times of political upheaval, community accepted the measure without objections.

Smallpox vaccination is part of ‘medicalisation’, in that the first campaigns were for many inhabitants the only concrete and effective presence of the State. With the vaccines, new professionals emerged, and other sanitation or disinfection measures were considered for the prevention of other health problems. In 1941, diphtheria vaccination was made mandatory.^82^

In 1977, the UN created the Expanded Programme on Immunization at the international level, which Argentina adhered to in 1978 with four vaccines, and progressively expanded to include other vaccines.^83^ When smallpox was eliminated, the vaccination schedule included vaccines for diphtheria, measles and poliomyelitis, and the epidemiologic surveillance services originally designed for smallpox eradication were forged into an infrastructure for control of these other diseases.^84^ Later, indications were given to destroy the virus stock since the disease could no longer be transmitted or spread by any means (animals or person to person), and the WHO was responsible for a strict supervision of the laboratories with reservoirs of the virus.^85^ This last point embodies how complex issues related to highly infectious and Epidemic diseases can be involved with multiple political interests.

## Footnotes

1 For the analysis carried out by official organizations, see Bichat Rodrigues , ‘Smallpox Eradication in the Americas’, Bulletin of the Pan American Health Organization, 9, 1 (1975), 53–68 167890; *L’Eradication Mondiale de la Variole, Rapport final de la Comission Mondiale pour la Certification de la Eradication de la Variole* (Genève: Organisation Mondiale de la Santé, 1980); ‘Small Pox Is Dead’, *World Health, the Magazine of the World Health Organization*, 1980, 1–40; Frank Fenner *et al.*, *Smallpox and Its Eradication* (Geneva: Word Health Organization, 1988). For other contributions on this subject, refer to Marcos Cueto and Steven Palmer, *Medicine and Public Health in Latin America: A History* (New York: Cambridge University Press, 2015); Anne-Emanuelle Birn , ‘Sueños y pesadillas de la erradicación de la viruela: reflexiones críticas para la salud global’, in *La erradicación y el control de las enfermedades infecciosas,* coords. María Isabel Porras Gallo , María José Báguena Cervellera , Mariano Ayarzagüena Sanz and Noelia María Martín Espinosa (Madrid: Catarata, 2016), 39–49 ; María Isabel Porras Gallo and Rosa Ballester Añón, ‘Luces y sombras de la erradicación de las enfermedades infecciosas: interés historiográfico y reflexión para la salud pública actual’, in *La erradicación y el control de las enfermedades infecciosas,* coords. María Isabel Porras Gallo, María José Báguena Cervellera, Mariano Ayarzagüena Sanz and Noelia María Martín Espinosa (Madrid: Catarata, 2016), 19–38; Christine Holmberg , Stuart Blume and Paul Greenough (eds), The Politics of Vaccination. A Global History (Manchester: Manchester University Press, 2017).

2 For a study on epidemiology, Latin America and global history, see Mariola Espinosa , ‘Globalizing the History of Disease, Medicine, and Public Health in Latin America’, Isis, 104, 4 (2013), 798–806 2478349610.1086/674946; James Jr. Webb , ‘Historical Epidemiology and Global Health History’, História, Ciências, Saúde – Manguinhos, 27 (2020), 13–28 3299705510.1590/S0104-59702020000300002; Anne-Emanuelle Birn , ‘How to Have Narrative-Flipping History in a Pandemic: Views of/from Latin America’, Centaurus, 62 (2020), 354–69, https://doi.org/10.1111/1600-0498.12310.

3 The Crude Death Rate was 31.9/1 000 between 1870 and 1875; 28.9/1 000 between 1895 and 1990; 15.1/1 000 between 1920 and 1925; 11.3/1 000 between 1940 and 1945; 8.8/1 000 between 1960 and 1965 and 8.5/1 000 between 1980 and 1985. See Carlos Grushka, ‘Casi un siglo y medio de mortalidad en la Argentina’, *Revista Latinoamericana de Población*, 15 (2014), 109.

4 Juan Carlos Tedesco and Alejandra Cardini, ‘Educación y sociedad: proyectos educativos y perspectivas futuras’, in *Población y bienestar en la Argentina del primero al segundo Centenario. Una historia social del Siglo XX. Tomo II,* Comp. Susana Torrado (Buenos Aires: EDHASA, 2007), 462.

5

Anne Marie Moulin , ‘Prémières vaccines, premières réticenses’, Pour la Science, 264 (1999), 13–15 .

6

Emilio Coni , ‘La viruela en Buenos Aires’, La Semana Médica, 49 (1917), 629 .

7 For studies on Mexico, see Ana María Carrillo , ‘Vaccine production, national security anxieties and the unstable state in nineteenth- and twentieth-century Mexico’, in Christine Holmberg , Stuart Blume and Paul Greenough (eds), The Politics of Vaccination. A Global History, (Manchester: Manchester University Press, 2017), 121–47; for studies on Brazil, Gilberto Hochman, ‘Vacinação, varíola e uma cultura da imunização no Brasil’, *Ciência & Saúde Colectiva*, 16,2 (2011), 375–86; Jaime Benchimol , ‘Yellow fever vaccine in Brazil: fighting a tropical scourge, modernising the Nation *’,* in Christine Holmberg , Stuart Blume and Paul Greenough (eds), The Politics of Vaccination. A Global History (Manchester: Manchester University Press, 2017), 174–208 ; Cueto and Palmer, *op. cit.* (note 1).

8
Bhattacharya, Sanjoy and Carlos Eduardo Ávila Pereira Campani Re-assessing the Foundations: Worldwide Smallpox Eradication, 1957–67 Medical History (2020) Jan;64(1):71–93. doi: 10.1017/mdh.2019.77.

9

Sheldon Watts , Epidemics and History: Disease, Power, and Imperialism (New Haven: Yale University Press, 1999); James C. Riley , ‘Smallpox and American Indians Revisited’, Journal of the History of Medicine and Allied Sciences, 65 (2010), 445–772021973010.1093/jhmas/jrq005.

10 Various types of smallpox have been identified; during much of the nineteenth century, two main forms were considered: discrete or minor smallpox, which caused a mild infection, and confluent/haemorrhagic or major smallpox, with lower chances of survival and greater side effects. In the twentieth century, a distinction was established between smallpox and alastrim, considering the latter to be less virulent than the former. See also *L’Eradication Mondiale de la Variole, op. cit.* (note 1).

11 Juan José Díaz, ‘Profilaxis de la viruela’ (unpublished PhD thesis: Universidad de Buenos Aires, 1876).

12 Carlos Baron De Finck, ‘Disertación de la viruela y su tratamiento’ (unpublished PhD thesis: Universidad de Buenos Aires, 1855).

13 Act 4202 (Argentina: 1903–4).

14

Diego Armus , The Ailing City. Health, Tuberculosis and Culture in Buenos Aires, 1870–1950 (Durham and London: Duke University Press, 2011).

15 Alejandro Amoretti, ‘Vacuna e inoculación vaccínica*’* (Tesis Inaugural: Buenos Aires, 1886).

16 For more information on the anti-vaccination movement in Great Britain, see Nadja Durbach , Bodily Matters: The Anti-Vaccination Movement in England, 1853–1907 (Durham and London: Duke University Press, 2005).

17

María Silvia Di Liscia , ‘Marcados en la piel. Vacunación y viruela en Argentina (1870–1910)’, Cadernos de Saúde Pública, 16, 2 (2011), 409–2210.1590/s1413-8123201100020000521340317.

18 Amoretti, *op. cit.* (note 15).

19 Provinces existing before 1884 are called ‘historical’ provinces: Salta, Jujuy, Catamarca, La Rioja, Tucumán, Mendoza, San Juan, San Luis, Córdoba, Santiago del Estero, Santa Fe, Entre Ríos, Corrientes and Buenos Aires. In 1884, after bloody military campaigns, the National Territories were organized on lands previously under control of the indigenous ethnic groups. These Territories were mostly provincialized throughout the twentieth century and were called the National Territory of La Pampa, Neuquén, Río Negro, Chubut, Santa Cruz, Tierra del Fuego, the Andes, Misiones, Chaco and Formosa.

20 For the complete list of authorities and their main efforts, see Carolina Biernat, ‘El proceso de centralización del Departamento Nacional de Higiene (1880–1914)*’, in La salud pública y la enfermería en Argentina,* dir. Carolina Biernat, Juan Manuel Cerdá and Karina Inés Ramacciotti (Buenos Aires: UNQUI, 2015), 47–83. A summary of the health policies can be found in Juan Carlos Veronelli and Magalí Veronelli Correch, *Los orígenes de la salud pública en Argentina,* Vol. II (Buenos Aires: Organización Panamericana de la Salud, 2004).

21 Díaz, *op. cit.* (note 11); Amoretti, *op. cit.* (note 15).

22 The institution changed its name and expanded its functions; it became the Instituto Nacional Carlos Malbrán in 1941, the Instituto Nacional de Microbiología in 1957 and the Instituto Nacional de Microbiología Dr. Carlos Malbrán in 1967.

23 Alfredo Larguía, ‘La vacuna en la República Argentina’ (unpublished PhD thesis: Universidad Nacional de Buenos Aires, 1902).

24
*Ibid.*

25
*Ibid.,* 52.

26 José Penna and Horacio Madero, *La administración sanitaria y la asistencia pública de la ciudad de Buenos Aires,* Vol. II (Buenos Aires: Imprenta, Litografía y Encuadernación G. Kraft, 1910), 96.

27 Veronelli & Veronelli Correch, *op. cit.* (note 20).

28 Fernando Alvarez, ‘La viruela y la vacuna en la República Argentina’, *El Monitor de la Educación Común*, 530 (1917), 101–5.

29

José Penna and Antonio Restagnio , Atlas sanitario argentino (contribuciones para su estudio) (Buenos Aires: Ministerio del Interior, 1916).

30 ‘Viruela’, *Boletín de la Oficina Sanitaria Panamericana,* 16 (Agosto 1937), 760–71.

31 For more information on PAHO, see Cueto and Palmer, *op. cit.* (note 1).

32 ‘Epidemia de viruela’ *Santa Fe,* 10 de febrero de 1923.

33 ‘La viruela en las Américas’, *Oficina Sanitaria Panamericana*, 2, 304 (1924).

34 ‘Progresos realizados en higiene e informes sanitarios’, *Boletín de la Organización Panamericana de la Salud*, 7 (Enero 1928), 273–80.

35 ‘Viruela’, *Boletín de la Oficina Sanitaria Panamericana,* 10 (Octubre 1931), 1328–31.

36 ‘Epidemia de viruela’ *El Orden, (*7 de octubre de 1935.

37 ‘Viruela’, *Boletín de la Oficina Sanitaria Panamericana,* 15 (Julio 1936), 679–85.

38 A key book which reviews this period is Gregorio Araoz Alfaro, *Por nuestros niños y por las madres: protección, higiene y asistencia social* (Buenos Aires: Cabaut, 1936). See a discussion of these topics in Susana Belmartino, *La atención médica argentina en el Siglo XX* (Buenos Aires: Sudamericana, 2005).

39 ‘Viruela’, *op. cit.* (note 37).

40 ‘La sanidad en Argentina’*, Boletín de la Oficina Sanitaria Panamericana,* 16 (Junio 1937), 509–22.

41 ‘Argentina’, *Boletín de la Oficina Sanitaria Panamericana,* 18 (Agosto 1939), 875–9.

42 ‘Viruela’, *op. cit.* (see note 30).

43 Since 1914, municipal slaughterhouses provided (free of charge) glands and other orthotherapeutic products to elaborate these medicines. See Viviana Román, ‘El sector farmacoquímico en la Argentina. Orígenes, desarrollo y acción estatal’, in *Arturo Oñativia y la Ley de Medicamentos, Cuadernos del Instituto de Salud*, no. 4, Viviana Román *y* Ernesto Salas (Florencio Varela: UNAJ, 2020), 11–28. See also ‘*La sanidad en Argentina*’, *Boletín de la Oficina Sanitaria Panamericana*, 16, 6 (Oficina Sanitaria Panamericana, 1937), 509–22.

44 Anne Marie Moulin, ‘Les déterminants de la politique vaccinale’, *ADSP,* 71 (Juin 2010), 14–16.

45 ‘Viruela’, *op. cit.* (note 30).

46 See ‘Viruela’, *op. cit.* (note 35) to know more on the studies from Institut Pasteur in Paris and Saigon. To know about the studies carried out in Germany and the United States, see ‘Viruela’, *op. cit.* (note 37).

47 Miguel Sussini, ‘La sanidad en Argentina’, *Boletín de la Oficina Sanitaria Panamericana,* 18 (Junio 1939), 509–15.

48

Pablo Kreimer and Hugo Ferpozzi , ‘De Milstein a la bioinformática. Emergencia y desarrollo de la biología molecular en Argentina’, in Pablo Kreimer (ed.), Contra viento y marea. Emergencia y desarrollo de campos científicos en la periferia, Argentina, Segunda Mitad del Siglo XX (Buenos Aires: CLACSO, 2016), 105–45.

49 Ramón Carrillo, emblematic sanitarist and prime minister of the area, considered that smallpox had already been ‘eliminated’ thanks to the battle fought with compulsory vaccination. With war metaphors – very common in the medical jargon of that time – smallpox was described as a plague already overcome, thanks, in part, to the compulsoriness of the measure. This indicator (compulsoriness of the vaccine) was considered key when it came to assess the health policies of a nation. Thus, just to mention an example of this assessment, countries where the smallpox vaccine was obligatory were given a score of 100 points, whereas those which did not enforce this measure were given 0 points. See Ramón Carrillo , Política sanitaria argentina (Buenos Aires: Ministerio de Salud de la Nación, 1949).

50

Karina Inés Ramacciotti , ‘Hospitales públicos *y* campañas sanitarias, (1945–1955)’, in La salud pública *y* la enfermería en Argentina, dir. Carolina Biernat , Juan Manuel Cerdá and Karina Inés Ramacciotti (Buenos Aires: UNQUI, 2015), 123–68.

51 ‘Viruela’, *El Litoral,* 17 de noviembre de 1950.

52 Julio Hansen, ‘Vacunación antivariólica’, *Revista de Educación Sanitaria*, 5–6 (1954), 10.

53 ‘Peligro de epidemia de viruela’, *El Litoral,* 5 de julio de 1957.

54 Ramacciotti, *op. cit.* (note 50).

55 The WHO was established in 1948 and since its inception, member states were repeatedly urged to take all measures at their disposal to control smallpox. In 1953, the first Director-General, Dr Brock Chisholm, made an unsuccessful attempt to persuade the Assembly to undertake a global initiative for an eradication programme. Five years later, the Soviet delegate, Viktor Zhdanov, convinced the Assembly to accept it, but only minimal funding was provided. Although in 1967 the disease was eliminated from around 30 countries in Asia, Africa and South America, in the Indian subcontinent and most of the countries in sub-Saharan Africa, there were no major changes until the end of the seventies. See Fenner *et al., op. cit.* (note 1); Rodrígues, *op. cit.* (note 1).

56 These relationships are woven beyond the traditional participants (businessmen, secret agents, military and armed forces or diplomatic organizations) and encompass others from a wide range of activities (cultural and social). Health matters could be included in these relationships as they had been considered before by the PAHO, among other agencies. See Benedetta Calandra and Marina Franco , ‘Desafíos y límites para una nueva mirada de las relaciones interamericanas’, in Benedetta Calandra and Marina Franco (eds), La guerra fría cultural en América Latina. Desafíos y límites para una nueva mirada de las relaciones interamericanas (Buenos Aires: Biblios, 2012), 35–50 .

57 ‘Sexta reunión de los Ministros de Salud de los países de la Cuenca del Plata’, *Boletín de la Oficina Sanitaria Panamericana,* 66 (Marzo 1969), 258–62.

58 The data were intended to help identify the infected people and the source of infection. The information, including date, probable origin of the outbreak, had to be immediately submitted to the Ministry or Asistencia Pública (Public Assistance), responsible for providing medical attention, indicating isolation for the infected individuals and decide on the corpses. The fine ranged from $500 to $10 000 (national currency), a hefty sum at the time, and included suspension from professional practice. See Law 15.465 (Argentina: 1960).

59 Campins, Mónica y Ana Pfeiffer Campins and Pfeiffer, ‘La industria farmacéutica argentina (1958–2010)’, *Anuario CEEED*, 9 (2017), 91–133.

60 Nobel Prize in 1984 for his research on monoclonal antibodies.

61 Kreimer, Pablo and Hugo Ferpozzi. ‘De Milstein a la bioinformática. Emergencia y desarrollo de la biología molecular en Argentina’. in: Kreimer, Pablo (Ed.). Contra viento y marea. Emergencia y desarrollo de campos científicos en la periferia, Argentina, Segunda Mitad del Siglo XX. Buenos Aires: CLACSO, 2016, p. 105–145. Kreimer and Ferpozzi, ‘De Milstein a la bioinformática’, in Kreimer (ed.), *Contra viento y marea. Argentina, Segunda Mitad del Siglo XX.* According to the authors, as a result of the political and scientific turmoil in the country, advances derived more from work on the sanitarist approach than from research in new fields.

62 ‘With home vaccination, the actual provincial campaign against smallpox began’, *El Litoral,* 27 October 1960.

63
*Ibid.*

64 Censo Nacional de Población (National Population Census), *Tomo IV* (Zona Central: Córdoba-Santa Fe, 1960).

65 Bhattacharya and Ávila Pereira Campani, *op. cit.* (note 8).

66 Organisation Mondiale de la Santé, *L’Eradication y* Cueto and Palmer, *op. cit.* (note 1).

67 For the study of viral DNA, the different species of Orthopoxvirus (Mokeypox, Teterapox, Variola major, Alastrim, Vaccinia Lister and Vaccinia Venezuela) were compared through serological tests. Fenner *et al., op. cit.* (note 1), 94.

68
*Ibid.*, 544.

69 Rodrigues, *op. cit.* (note 1). Fenner *et al., op. cit.* (note 1).

70
*Ibid.*, 560, 564.

71
*Ibid.*, 584.

72 Rodrigues, *op. cit.* (note 1).

73 Fenner *et al., op. cit.* (note 1). For a detailed analysis of Brazil, see Cueto and Palmer, *op. cit.* (note 1).

74 Rodrigues, *op. cit.* (note 1).

75 By way of comparison, in the same period in Bolivia, much lower figures were documented for a population in similar conditions (between 400 000 and 235 250 doses). Bolivia used laboratories in Argentina and Brazil for the diagnosis of smallpox in the late 1960s, and no longer turned to the centres in North America established by the programme. Rodrigues, *op. cit.* (note 1). Fenner, Henderson, Arita, Jezek & Ladnyi, *smallpox eradication.*

76 Rodrigues, *op. cit.* (note 1).

77 ‘Protegerse contra la viruela no es sólo un derecho sino un deber’, *El Litoral,* 10 de septiembre de 1973.

78 Furthermore, the economic factors indicated by Arita, one of the WHO specialists in 1980, must have influenced: ‘The cessation of vaccination will not only save thousands of patients who would otherwise have suffered from complications but will also save the world community some $1 000 million a year’. Isao Arita, *Can We Stop Smallpox Vaccination?* (Geneva: World Health, the Magazine of the World Health Organization, 1980), 27. See also Ley 22.109 (Argentina, 22 November 1979); Poder Ejecutivo Nacional, *Boletín Oficial* (Buenos Aires: 30 November 1979), http://servicios.infoleg.gob.ar/infolegInternet/anexos/230000-234999/231268/norma.htm (accessed on 20 December 2020).

79 Birn, *op. cit.* (note 1); Cueto and Palmer, *op. cit.* (note 1).

80 Román, Viviana. ‘El sector farmacoquímico en la Argentina, in Román, Viviana y Salas, Ernesto, Arturo Oñativia y la Ley de Medicamentos, Florencio Varela: UNAJ, Cuadernos del Instituto de Salud N°4, 2020, p.11–28.

81 Such was the slogan used in 1980. See ‘Small Pox Is Dead’, *op. cit.* (note 1).

82 National Law No. 12670. Official Bulletin, 8 May 1941. Regulations: Decree Law No. 104.166, 28 October 1941.

83 In 1997, the triple viral vaccine (against measles, rubella and mumps) was added; in 1998, the conjugate vaccine Haemophilus influenzae type b (included in the combination vaccine [DTP, Hib]); in 2000, the vaccine against hepatitis B (HB) and in 2005, the vaccine against hepatitis A. In 2012, the country had 16 vaccines. See *Vacunas: el Derecho a la Prevención* (Buenos Aires: Ministerio de Educación de la Nación y Ministerio de Salud de la Nación, 2012).

84 Rodrigues, *op. cit.* (note 1).

85
*Éradication de la variole, destruction des stocks de virus variolique Rapport du Secrétariat* (Géneve: OMS, 2011).

